# Rapid Generation of TCR and CD8αβ Transgenic Virus Specific T Cells for Immunotherapy of Leukemia

**DOI:** 10.3389/fimmu.2022.830021

**Published:** 2022-04-29

**Authors:** Gagan Bajwa, Caroline Arber

**Affiliations:** ^1^Center for Cell and Gene Therapy, Baylor College of Medicine, Houston Methodist Hospital and Texas Children’s Hospital, Houston, TX, United States; ^2^Department of Oncology UNIL CHUV, Lausanne University Hospital (CHUV), University of Lausanne (UNIL), and Ludwig Institute for Cancer Research Lausanne Branch, Lausanne, Switzerland

**Keywords:** immunotherapy, virus-specific T cells, cytokine capture, transgenic TCR, transgenic CD8, engineered T cells, interferon-gamma

## Abstract

**Background:**

Virus-specific T cells (VSTs) are an attractive cell therapy platform for the delivery of tumor-targeted transgenic receptors. However, manufacturing with conventional methods may require several weeks and intensive handling. Here we evaluated the feasibility and timelines when combining IFN-γ cytokine capture (CC) with retroviral transduction for the generation of T cell receptor (TCR) and CD8αβ (TCR8) transgenic VSTs to simultaneously target several viral and tumor antigens in a single product.

**Methods:**

Healthy donor peripheral blood mononuclear cells were stimulated with cytomegalovirus (CMV) and Epstein-Barr-Virus (EBV) peptide mixtures derived from immunogenic viral proteins, followed by CC bead selection. After 3 days in culture, cells were transduced with a retroviral vector encoding four genes (a survivin-specific αβTCR and CD8αβ). TCR8-transgenic or control VSTs were expanded and characterized for their phenotype, specificity and anti-viral and anti-tumor functions.

**Results:**

CC selected cells were efficiently transduced with TCR8. Average fold expansion was 269-fold in 10 days, and cells contained a high proportion of CD8+ T central memory cells. TCR8+ VSTs simultaneously expressed native anti-viral and transgenic anti-survivin TCRs on their cell surface. Both control and TCR8+ VSTs produced cytokines to and killed viral targets, while tumor targets were only recognized and killed by TCR8+ VSTs.

**Conclusions:**

IFN-γ cytokine capture selects and activates CMV and EBV-specific memory precursor CD8+ T cells that can be efficiently gene-modified by retroviral transduction and rapidly *ex vivo* expanded. Our multi-specific T cells are polyfunctional and recognize and kill viral and leukemic targets expressing the cognate antigens.

## Introduction

Adoptive transfer of virus specific T cells (VSTs) rapidly restores antiviral immunity and prevents or treats viral infections after allogeneic hematopoietic stem cell transplantation (HSCT) (1). VSTs are both safe and effective when manufactured from the original stem cell donor or from unrelated partially HLA matched third party healthy donors (1, 2), setting the stage for their use as a cellular therapy platform for the delivery of engineered receptors targeting tumor-associated antigens (recently reviewed in (3)). Indeed, leukemia targeted chimeric antigen receptors (CARs) such as CD19-CARs in B-cell acute lymphoblastic leukemia (B-ALL) or T cell receptors (TCRs) targeting Wilms Tumor 1 (WT1) or the minor histocompatibility antigen HA-1H in acute myeloid leukemia (AML) have been expressed in VSTs and infused to patients post-transplant (4–9). Safety and some efficacy was demonstrated with CD19-CAR-modified VSTs produced from the stem cell donor (4–6), while feasibility and efficacy with TCR-modified VSTs was variable among studies (7, 8).

Manufacturing of engineered VSTs is challenging and operator intensive. Certain steps are performed in open systems such as flow cytometry-based sorting (7), or require knowledge of the targeted epitope such as streptamer-selection (8). Other processes require live Epstein Barr Virus (EBV) for the generation of autologous lymphoblastoid cell lines (4–6), several types of viral vectors for the transduction of antigen presenting cells and transduction of VSTs (3, 6), and prolonged *ex vivo* culture over several weeks (4–7).

For broader applicability of such multi-antigen targeted therapies, the complexity of the production processes needs to be reduced (recently reviewed in (10)). Here, we investigated at small scale if Interferon-γ (IFN-γ) cytokine capture (CC) selected virus-specific memory T cells from healthy donors are sufficiently enriched and activated to directly proceed to retroviral transduction introducing an HLA-A*02:01 restricted survivin targeted TCR (11) in combination with CD8αβ (TCR8) to redirect VSTs to a broad tumor-associated antigen (12, 13). We have previously demonstrated that the incorporation of CD8αβ as a transgene restores anti-viral activity of TCR transgenic VSTs, and redirects CD4+ T cells to the class I restricted cognate antigen (12, 13). CC is attractive because it is compatible with fully closed production of VSTs independently of donor HLA and can select T cells with diverse TCR repertoires recognizing various immunogenic epitopes (14–16). Now we show that enrichment and activation of anti-viral memory T cells by CC followed by retroviral transduction reduces manufacturing time by 7-10 days, reduces the overall complexity of the process, and yields cells with simultaneous anti-viral and anti-tumor activity.

## Materials and Methods

### Cell Lines

BV173 and K562 cell lines were obtained from the German Cell Culture Collection (DSMZ) or the American Type Culture Collection (ATCC), respectively, and maintained in complete RPMI 1640 media (Hyclone, Thermo Scientific) supplemented with 10% or 20% fetal bovine serum (FBS, Hyclone), 1% penicillin-streptomycin (Gibco) and 1% glutamax (Gibco) (13). 293T cells (ATCC) were maintained in complete IMDM media (Hyclone) containing 10% FBS, 1% penicillin-streptomycin, and 1% glutamax.

### Healthy Donor Buffy Coats

Buffy coats from CMV seropositive de-identified healthy human volunteers were procured from the Gulf Coast Regional Blood Center (Houston, TX, USA). HLA-A2 status was determined by FACS analysis and HLA-A2 positive donors were selected for the experiments.

### Generation of Retroviral Vectors and Supernatant

**Figure 1:**
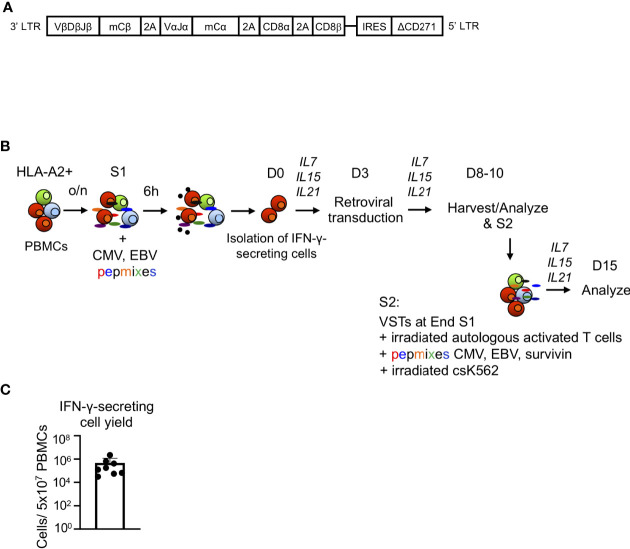
Generation of TCR8 transgenic VSTs by IFN-γ cytokine capture and retroviral transduction. **(A)** Schematic of the retroviral vector containing the survivin-specific TCR with murine constant regions (mCα, mCβ), CD8αβ and a truncated selectable marker (ΔCD271). **(B)** Steps involved in the production of transgenic VSTs using IFN-γ CC, retroviral transduction and cell expansion. S1: first stimulation, S2: second stimulation (optional). **(C)** Total yield of IFN-γ captured cells from 5x10^7^ PBMCs after immunomagnetic separation, n=8 donors, mean ± SD.

The design of the retroviral vector encoding the survivin-specific (s24) TCR and CD8αβ has been described previously (**Figure 1A**) (11–13). Retroviral supernatant was prepared by transient co-transfection of 293T cells with RD114 and Pegpam plasmids and the SFG vector containing the genes of interest (11).

### Generation of Gene Modified Virus-Specific T Cells Using IFN-γ Cytokine Capture and Retroviral Transduction

PBMCs were isolated from buffy coats using density gradient centrifugation by Lymphoprep (Accurate Chemical and Scientific Corporation), resuspended in T cell complete media (1:1 mixture of RPMI 1640 and Click’s media, Hyclone, supplemented with 10% FBS, Hyclone, 1% penicillin-streptomycin, 1% glutamax) and rested overnight at 37°C (10^7^ PBMCs per 2 ml in complete media/well in 24-well plate). After incubation, PBMCs were stimulated with a mixture of CMV and EBV pepmixes (CMV pp65, CMV IE1, EBV LMP2, EBV BZLF1, EBV EBNA, 1 μg/ml, all from JPT Technologies) and HLA-A*02:01 restricted immunodominant peptides (CMV pp65 derived NLV: NLVPMVATV, immediate early EBV BRLF1-derived YVL: YVLDHLIVV, early EBV BMLF1-derived GLC, 1 μg/ml, all from Genemed Synthesis) for 6 h at 37°C. The combination of pepmixes and peptides was chosen based on antigen expression patterns of CMV and EBV infection in the post-transplant period (1). IFN-γ secreting cells were isolated using the IFN-γ secretion assay-cell enrichment kit (Miltenyi Biotech, #130-054-202) according to the manufacturer’s recommendations (**Figure 1B**). IFN-γ secreting cells were plated in complete media with IL7 (10 ng/mL, R&D Systems), IL15 (10 ng/mL, R&D Systems) and IL21 (30 ng/mL, R&D Systems), and the irradiated IFN-γ-negative fraction (30 Gy) was used as a feeder layer (0.02-0.5 x10^6^ IFN-γ captured cells per 0.5 x10^6^ feeder cells per well in 24-well plate). After 3 days, IFN-γ cytokine captured VSTs were harvested and transduced with retroviral supernatant encoding the survivin-specific αβTCR and CD8αβ (TCR8, **Figure 1A**) on retronectin coated plates or exposed to retronectin coated plates without viral particles (for non-transduced VSTs). VSTs were expanded for 5-7 days in the presence of cytokines IL7 (10 ng/mL, R&D Systems), IL15 (10 ng/mL, R&D Systems) and IL21 (30 ng/mL, R&D Systems). A second stimulation (S2) was performed to evaluate the further expansion potential of the engineered VSTs. S2 was performed with CMV/EBV pepmix/peptide and HLA-A*02:01 restricted survivin peptide LMLGEFLKL (the cognate antigen of the transgenic TCR, Genemed synthesis) pulsed irradiated (40 Gy) autologous activated T cells (previously activated on OKT3/anti-CD28 coated plates) and irradiated (100 Gy) K562cs cells (K562 cells engineered to express CD80, CD83, CD86, 41BBL and CD32) at a ratio of 1:1:5 (**Figure 1B**) in G-Rex gas permeable culture devices (WilsonWolf, Saint Paul, USA) as previously described (17).

### Immunophenotyping

For evaluation of cell surface marker expression, cells were stained with FITC-, phycoerythrin (PE-), allophycocyanin (APC), V450-, PerCP, APC-AF750 or Krome orange-conjugated antibodies (Abs) against CD4, CD8, CD45RO, CD62L, CCR7, CD45RA, CD56, TCRγδ, CD271, CD19, 7-AAD (BD Biosciences), murine TCRβ constant region (ebiosciences), NLV pentamer (Proimmune) or survivin LML dextramer (Immudex) for 30 min at 4°C. The degranulation assay was performed as described previously (13). Briefly, VSTs (10^6^) were treated with golgi-plug/brefeldin A (Invitrogen) and CD107a/b VioBlue (BD Biosciences) followed by appropriate stimulations: CMV/EBV specific viral pepmixes or single peptide (pp65, IE1, LMP2, BZLF1, EBNA1, GLC, 1 μg/ml), survivin-specific LML peptide, viral pepmixes/peptide plus LML peptide, PMA (25 ng/ml)/Ionomycin (1 μg/ml) or control Influenza matrix protein GIL (GILGFVFTL, Genemed synthesis) peptide (negative control) for 4 h at 37°C. Intracellular staining (ICS) was performed using anti-human IFN-γ-FITC and TNF-α-PE (BD Biosciences) antibodies. The samples were acquired on a FACS Canto with BD FACSDiva software, and analysis was performed using FlowJo software (Tree Star Inc.).

### IFN-γ ELISpot

For quantification of IFN-γ producing cells by ELISpot, VSTs (10^5^ per well) were plated in triplicates and stimulated with individual pepmixes/peptides (1 µg/ml) or cell lines (BV173 or K562) at 1:1 ratio (10^5^ cells per well) or media alone. Plates were incubated at 37°C/5% CO_2_ over-night and developed. Spot Forming Units (SFUs) were enumerated by ZellNet.

### Co-Culture Assay and Cytokine Detection

To determine the anti-tumor function, VSTs and BV173 cells were co-cultured at E:T ratio of 1:5 without exogenous cytokines. Supernatants from co-cultures were harvested after 24 h and were stored at -80°C for cytokine analysis. After 3 days, residual VSTs and tumor cells were enumerated using CountBright Beads (Life Technologies) and FACS analysis. Cytokines were quantified in supernatants using the MILLIPLEX Human CD8+ T-cells Magnetic Beads Panel (EMD Millipore) and a Luminex 200 instrument (Luminex).

### ^51^Chromium Release Assay

*In vitro* short-term cytotoxicity of VSTs was assessed using a standard ^51^Cr-release assay as described previously (13). Briefly, autologous activated T cells (targets) were pulsed with the indicated peptides or pepmixes (1 μg/ml) and labeled with ^51^Cr for 1 h. VSTs and target cells were incubated at various ratios for 4 h. For controls, target cells were incubated in media alone or with 1% triton-X 100 (Sigma-Aldrich) to measure the spontaneous and the maximum release, respectively. The mean percentage of specific lysis of triplicate wells was calculated as follows: [(test counts – spontaneous counts)/(maximum counts − spontaneous counts)] x100%.

### Statistical Analysis

Descriptive statistics was used to summarize the data. Comparison between groups was made using student’s t-test or One-Way ANOVA whichever was appropriate. GraphPad prism 6 (GraphPad software, Inc., La Jolla, CA) or higher was used for statistical analysis. P values <0.05 were considered statistically significant.

## Results

### Rapid Generation of Transgenic VSTs by IFN-γ Cytokine Capture and Retroviral Transduction

**Figure 2:**
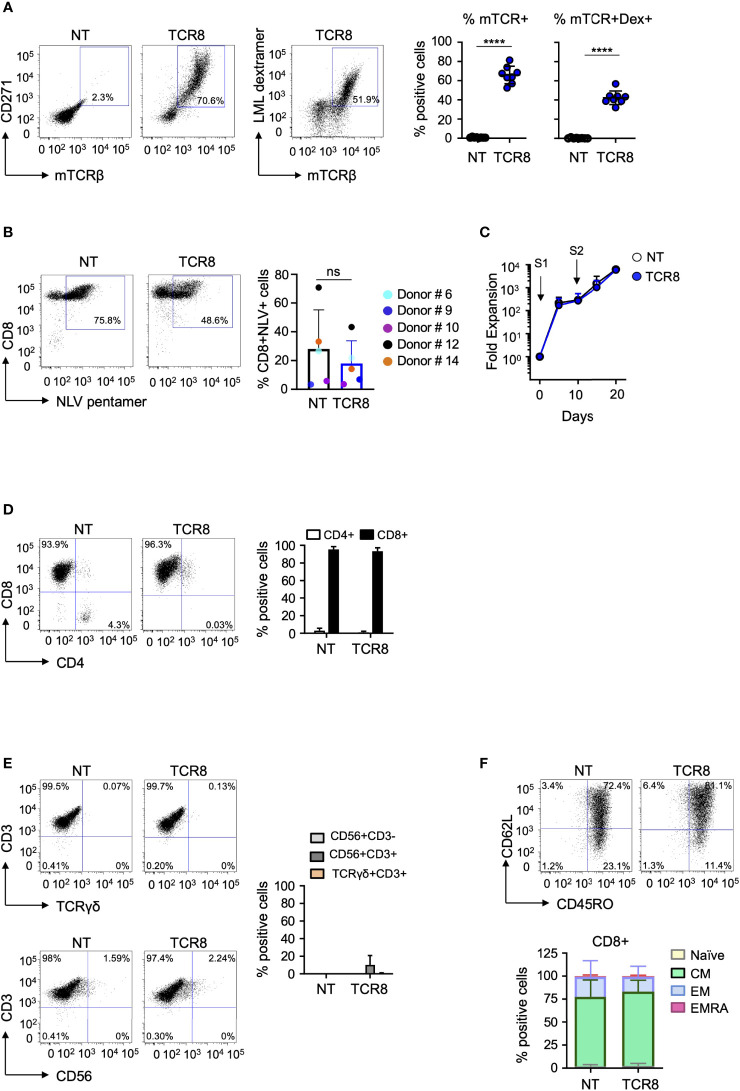
Phenotypic characterization and fold expansion of TCR8+ VSTs. **(A)** Representative FACS plots (left) and summary (right) of transduction efficiencies (mTCRβ, CD271) and transgenic TCR specificity [LML dextramer (Dex)]. NT: non-transduced. NT vs TCR8+, mean ± SD, ****p <0.0001, n=8 in both. **(B)** Representative FACS plots (left) and summary (right) of CMV-specific T cells in NT and TCR8+ VSTs. NT vs TCR8+, mean ± SD, n=5, p=ns. Dot color indicates individual donors. **(C)** Fold expansion of NT (open circles) and TCR8+ (blue circles) VSTs after first (S1) and second (S2) stimulation. **(D)** Distribution of CD4+ and CD8+ T cells, representative FACS plots (left) and summary (right) (gated on total live cells), mean ± SD, n=5. **(E)** Analysis of NK cells (CD56+CD3-), activated T cells (CD56+CD3+), or TCRγδ T cells (TCRγδ+CD3+) in NT and TCR8+ VSTs. Representative FACS plots (left) and summary (right) (gated on total live cells), mean ± SD, n=5. **(F)** Representative FACS plot (top) and summary (bottom) (gated on live CD3+CD8+ T cells) of memory phenotype of VSTs: naïve (T_N_), central memory (T_CM_), effector memory (T_EM_) and terminally differentiated (T_EMRA_) subsets characterized in NT and TCR8+ VSTs based on CD45RO and CD62L staining, n=5, mean ± SD. ns, not significant.

To rapidly generate genetically engineered TCR8+ VSTs from healthy donors, we used the IFN-γ cytokine capture selection system to enrich for CMV and EBV-specific memory T cells upon peptide stimulation of peripheral blood mononuclear cells, followed by retroviral transduction. The scheme of the retroviral vector (**Figure 1A**) and the cell isolation, transduction and expansion process (**Figure 1B**) is shown in **Figure 1** and is fully Good Manufacturing Practice (GMP) laboratory compatible. The average yield of IFN-γ secreting VSTs from 50x10^6^ PBMCs was 0.15x10^6^ (range 3.13x10^4^ – 2.2x10^6^, n=8, **Figure 1C**). VSTs isolated by IFN-γ CC were sufficiently activated by the isolation to directly proceed to retroviral transduction on day 3 (TCR8+ vs NT VSTs, %mTCR+ cells; 66±9% vs 0.8±0.4%, p<0.0001, mean±SD, n=8) (**Figure 2A**). TCR8+ VSTs efficiently bound the survivin-specific dextramer compared to non-transduced (NT) VSTs (TCR8+ vs NT VSTs, %mTCR+Dex+; 42±7% vs 0.3±0.4%, p<0.0001, mean±SD, n=8, **Figure 2A**). Both, TCR8+ and NT VST lines were enriched in NLV+ cells (TCR8+ vs NT VSTs; 18±15.8% vs 28±27%, mean±SD, p=ns, n=5), based on NLV-pentamer staining that is used to detect CMV specific cells within the product (**Figure 2B**). Non-transduced and TCR8+ VSTs expanded well with comparable fold expansions after first (S1) (NT: 294±267-fold, TCR8+: 269±285-fold) and second (S2) (NT: 6284±6646-fold, TCR8+: 5877±6682-fold) stimulation respectively (mean±SD, n=5, **Figure 2C**). Phenotypically, both NT and TCR8+ VSTs consisted predominantly of CD3+CD8+ T cells (NT: 95±3%, TCR8+: 93±3%, mean±SD, n=8) with low percentages of CD3+CD4+ T cells (NT: 3±3%, TCR8+: 1±1%, mean±SD, n=8) (**Figure 2D**). We found a complete absence of NK cells (CD3-CD56+) and TCRγδ+ T cells in our products. However, TCR8+ VSTs contained slightly increased frequencies of CD3+CD56+ activated T cells as compared to NT VSTs (NT vs TCR8+: 0±0 vs 10±10%, p=ns, n=8) (**Figure 2E**). The memory subset distribution in the CD3+CD8+ compartment revealed high proportions of central memory T cells (T_CM_) in both, NT and TCR8+ VSTs (NT vs TCR8+, naïve (T_N_) 1.9±1.9% vs 2.5±2.7%, T_CM_; 75±18% vs 81±12%, effector memory (T_EM_) 22±18% vs 16±11%, terminally differentiated (T_EMRA_) 0.8±1.2% vs 0.7±0.9%, p=ns, n=6) (**Figure 2F**). Thus, we show that the combination of IFN-γ capture and retroviral transduction can rapidly generate engineered T cell products with simultaneous anti-viral and anti-tumor specificities that are highly enriched in CD8+ T_CM_ cells.

### IFN-γ Capture TCR8+ VSTs React Against the Targeted Viral and Tumor Antigens

**Figure 3:**
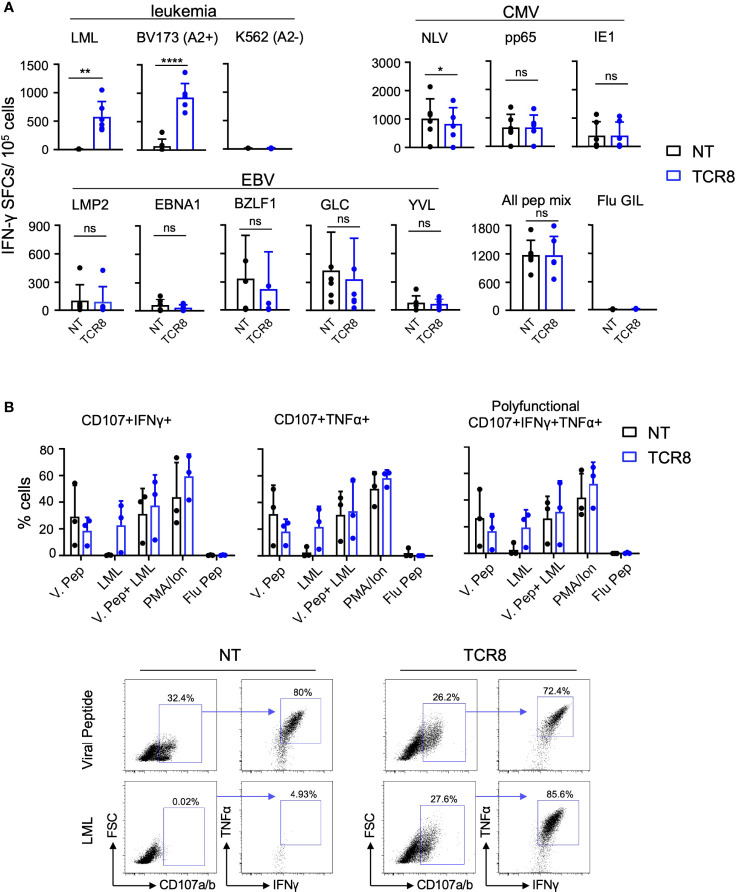
TCR8+ VSTs are multi-antigen specific for survivin and viral (CMV and EBV) antigens. **(A)** NT or TCR8+ VSTs after S1 were stimulated with viral (CMV and EBV) pepmixes/peptides, survivin peptide, or leukemia cell lines (BV173: HLA-A2*02:01+survivin+ or K562: HLA-A2-) for 24 h and IFN-γ spot forming cells (SFC) were measured by ELISPOT, n=6, mean ± SD: NT vs TCR8+, LML peptide: **p=0.003, BV173: ****p<0.0001, NLV peptide: *p=0.035, pp65, IE1, LMP2, BZLF, EBNA1, GLC peptide, YVL peptide: mean ± SD, p=ns, n=6. **(B)** NT or TCR8+ VSTs were stimulated with viral pepmixes, LML peptide, viral pepmixes plus LML peptide, PMA/Ionomycin (25 ng/ml) or irrelevant influenza GIL peptide (negative control) for 4 h and stained for degranulation (CD107a/b) and intracellular IFN-γ and TNF-α followed by FACS analysis. The percentage of cells expressing CD107a/b+/IFNγ+ (top left), CD107a/b+/TNFα+ (top middle) and (CD107a/b+/IFNγ+/TNFα+ (polyfunctional, top right) are shown. Dots, mean ± SD, n=3 donors. Representative FACS plots are shown on the bottom (gated on total live cells, then gated on CD107a/b+ cells) for NT (left) and TCR8 (right). ns, not significant.

Next, we assessed antigen specific function of NT and TCR8+ VSTs by IFN-γ ELISPOT and intracellular cytokine staining (ICS). As expected, TCR8+ but not NT VSTs produced IFN-γ in response to the cognate survivin peptide (LML) or the HLA-A*02:01+survivin+ leukemia cell line BV173, targeted by the transgenic TCR (IFN-γ SFCs NT vs TCR8+ VSTs; LML: 7.0±3.7 vs 577±268, p=0.003; BV173: 71.5±122 vs 925±246 p<0.0001, n=6, mean±SD) (**Figure 3A**, top left). Importantly, both NT and TCR8+ VSTs showed comparable anti-viral reactivities against CMV (pp65 and IE1 pepmix) and EBV (LMP2, EBNA1 and BZLF1 pepmixes, GLC and YVL peptides) antigens, while a small but significant reduction in NLV reactivity was observed with TCR8+ VSTs (**Figure 3A**). These results were corroborated by ICS where we found similar degranulation (CD107a/b), IFN-γ and TNF-α levels in NT and TCR8+ VSTs in response to viral antigens (**Figure 3B**). Again, the survivin derived LML peptide was only recognized by TCR8+ but not NT VSTs. Thus, IFN-γ capture TCR8+ VSTs are specific for and reactive against both the targeted tumor and viral antigens, and anti-viral reactivities are not altered by the transduction and forced expression of TCR8.

### IFN-γ capture TCR8+ VSTs Kill Viral and Tumor Targets *in vitro*

**Figure 4:**
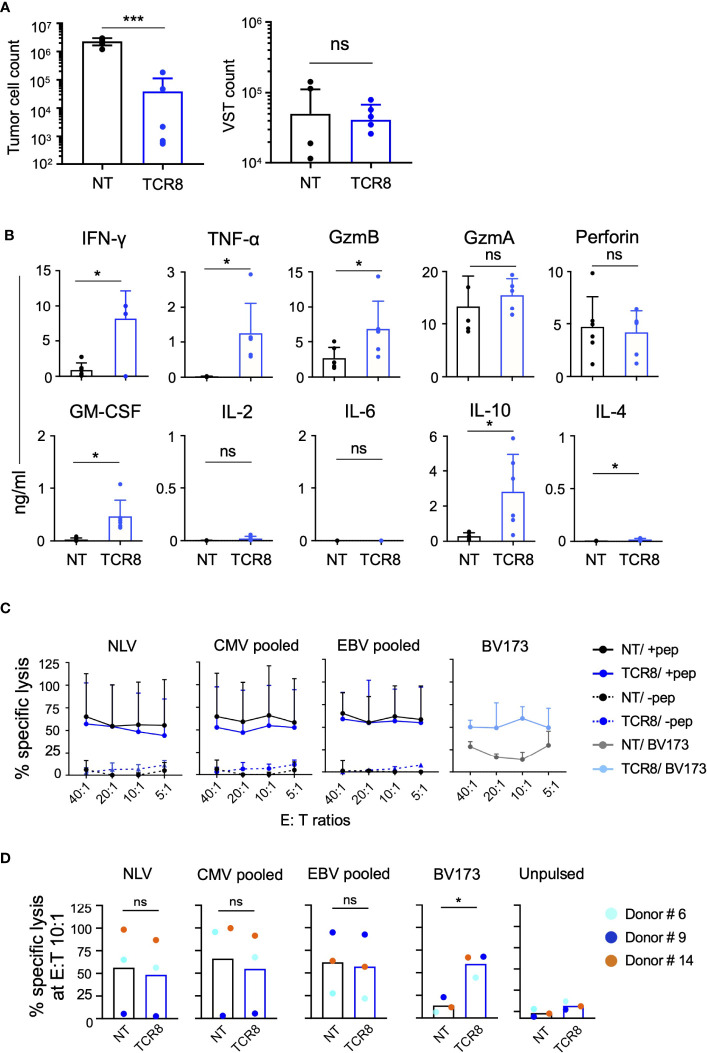
TCR8+ VSTs are cytotoxic *in vitro* against the cognate viral and tumor targets. **(A)** Co-culture of NT or TCR8+ VSTs with BV173 leukemia cells (HLA-A2*02:01+survivin+); E:T ratio 1:5. Residual BV173 cells (left) or VSTs (right) quantified by FACS on day 3, NT vs TCR8+, mean ± SD, Tumor cells: ***p=0.0004; VSTs: p=ns; n=6. **(B)** Cytokine quantification in supernatants after 24 hours of coculture, NT vs TCR8+, mean ± SD, IFN-γ, TNF-α, Granzyme (Gzm) B, GM-CSF, IL-10 and IL-4: *p<0.05, n=6. **(C)** Percent specific lysis of peptide/pepmix pulsed or unpulsed activated autologous T cells or BV173 cells by NT or TCR8+ VSTs in a 4-hour ^51^Cr-release assay at E:T ratios of 40:1, 20:1, 10:1, 5:1, mean ± SD, n=4. **(D)** Summary of percent specific lysis at E:T 10:1 ratio depicting individual donors (colored dots). NT vs TCR8+, mean ± SD, NLV peptide, CMV pooled, EBV pooled: p=ns; BV173 cells: *p=0.01, n=3, paired t-test. ns, not significant.

We next evaluated the cytotoxicity of NT and TCR8+ VSTs in co-cultures and in a 4-hour ^51^Chromium-release assay. When we co-cultured NT or TCR8+ VSTs with HLA-A*02:01+survivin+ BV173 leukemia cells, we observed significant killing of target cells by TCR8+ but not NT VSTs (residual tumor cell count NT vs TCR8+: 2.3±0.6x10^6^ vs 0.04±0.07x10^6^, p=0.0004, mean±SD, n=6) (**Figure 4A**, left). No difference in the VST counts at the end of the co-cultures was seen (**Figure 4A**, right). We also analyzed cytokine secretion and lytic granules present in the co-culture supernatant 24 hours after tumor challenge. TCR8+ VSTs produced significant amounts of T_H_1 cytokines including IFN-γ, TNF-α and IL-10 (NT vs TCR8+ VSTs: IFN-γ; 0.9±1.0 vs 8.1±4.0 ng/ml, p=0.017, TNF-α; 0.02±0.01 vs 1.2±0.9 ng/ml, p=0.017, IL-10; 0.3±0.2 vs 2.8±2.1 ng/ml, p=0.028, mean±SD, n=6) and cytolytic granules (GZMB NT vs TCR8+: 2.7±1.5 vs 6.8±4.0 ng/ml, p=0.02, mean±SD, n=6) (**Figure 4B**). We detected GZMA and perforin release into the supernatant of NT VSTs even though no cytotoxicity was observed. In a 4-hour ^51^Chromium-release cytotoxicity assay, we found that activated autologous T cells pulsed with viral peptides were efficiently lysed by both, NT and TCR8+ VSTs at various E:T ratios, while un-pulsed targets were not killed (**Figure 4C**, mean±SD, n=3 donors, each plated in technical triplicates). HLA-A*02:01+survivin+ BV173 leukemia cells were only killed by TCR8+ but not NT VSTs. Donor heterogeneity with regards to CMV and/or EBV reactivity was high, as illustrated in **Figure 4D**. For example, anti-viral specificity from donor #9 was almost exclusively directed against EBV and not against CMV, while the other two evaluated donors showed simultaneous responses against both viruses. The anti-leukemic activity conferred by the transgenic TCR was much more consistent across donors. Thus, we demonstrate that TCR8+ VSTs generated with our approach are cytotoxic and functional against both viral and tumor targets.

## Discussion

**Figure 5:**
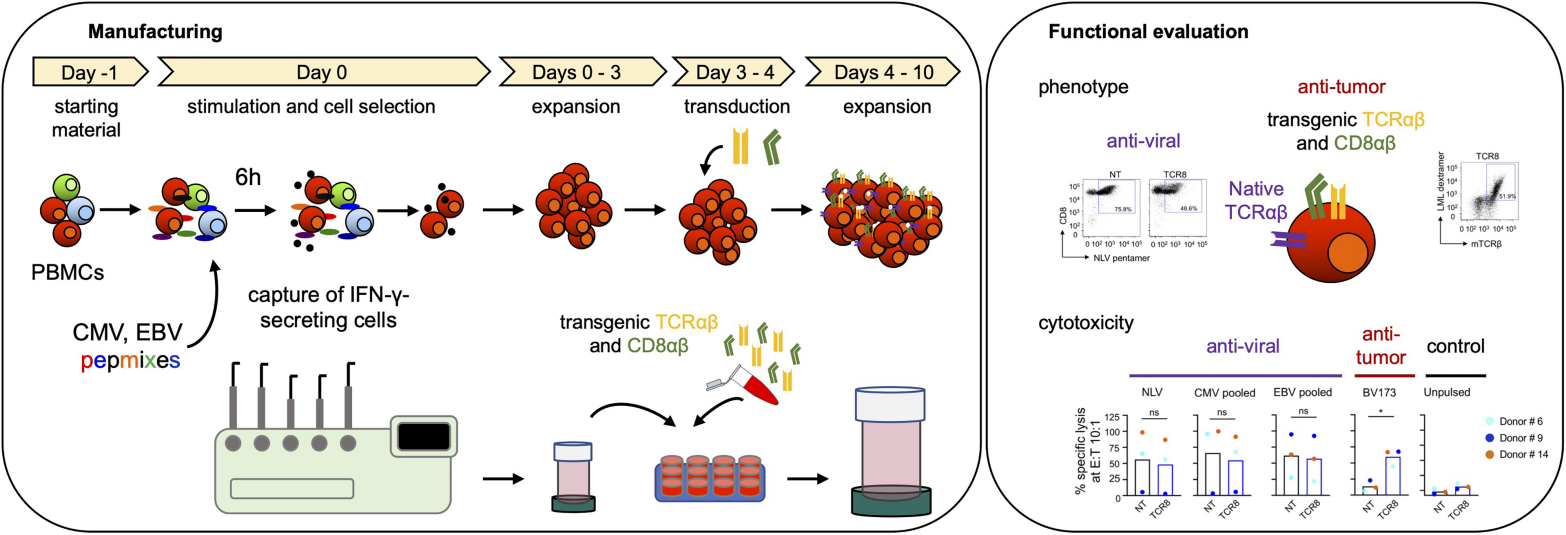
Schematic overview of transgenic virus-specific T cell manufacturing and functional evaluation.

Here we present an approach for the rapid generation of engineered human T cells with simultaneous anti-viral and anti-tumor activity (**Figure 5**). With the IFN-γ cytokine capture system we efficiently enriched and activated anti-viral memory CD8+ T cells that were directly amenable for retroviral transduction, significantly reducing the complexity of manufacturing compared to previously established processes. Transgenic co-expression of the CD8αβ co-receptor with the tumor targeted TCR ensured sufficient co-receptor availability for both endogenous anti-viral and transgenic anti-tumor TCRs. T cells generated with our approach efficiently recognized and killed both viral and tumor targets.

Our approach has significant advantages over other established processes for the production of engineered VSTs and allows considering moving to a semi-automated closed process. The most important advantages are that (1) no live virus is necessary for the production of lymphoblastoid cell lines, (2) viral antigen presentation and T cell activation is achieved with peptide pulsing of peripheral blood mononuclear cells and no additional generation of antigen presenting cells is necessary, (3) a one-step procedure is sufficient for T cell selection and activation that allows to directly proceed to gene-modification after a short culture period, (4) selection is performed with magnetic columns and does not require flow cytometry-based sorting, and importantly (5) the approach significantly reduces manufacturing time as well as the number of manipulations needed for product generation. IFN-γ captured VSTs can be potentially modified using non-viral gene delivery systems such as transposons or CRISPR/Cas9, that are more versatile and cost-effective (18). In addition, we plan to upscale the approach and reduce the need for open manufacturing steps. For example, cytokine capture as well as gene modification and cell expansion could be adapted to the capabilities of an automated closed manufacturing platform such as for example the CliniMACS Prodigy system (16, 19).

Nevertheless, we also identified some disadvantages, which include in our hands (1) the almost exclusive enrichment for CD8+ T cells, and (2) a high donor variability in T cell yield after the CC selection procedure. In fact, virus-specific CD4+ T cells play a key role in the development of long-lasting antiviral immunity by potentiating cytotoxic CD8+ T cell responses, by providing help to B cells for efficient and long-lasting antibody responses, and by direct cytotoxic effects (20–22). Upon adoptive transfer of VSTs to immunocompromised patients after HSCT, the CD4+ T cell compartment was instrumental for the development of long-lasting viral control (23). The lack of CD4+ T cell enrichment in our study may be due to the fact that our stimulation with the viral pepmixes was performed over 6 hours only, compared to previous literature where the stimulation lasted 16 hours (14, 24), a factor that needs to be evaluated in the future. The high variability in viral antigen-specific cell frequency is consistent with previous observations (14, 16, 24) and confirms the fact that circulating anti-viral memory T cell frequency varies over a broad range in different individuals.

VSTs are an interesting cell therapy platform for the development of allogeneic off-the shelf engineered T cell therapies. Several academic clinical trials have demonstrated safety and efficacy in controlling viral infections in immunocompromised patients after solid organ transplant or allogeneic HSCT with the infusion of third-party donor derived banked VSTs in partially HLA matched settings (25–30). Third party VST cell therapy is now on the way to commercialization. Because VSTs express a viral antigen restricted TCR repertoire, they did not produce significant graft-versus host disease in infused patients across studies. However, *in vivo* persistence was shorter when compared to VSTs derived from HLA matched donors [recently reviewed in (3)] indicating significant rejection by host T or NK cells. Recently, additional engineering strategies have been developed to confer resistance to rejection to the gene-modified VSTs (31, 32) which further enhances potential future applicability as a more general cell therapy platform.

In summary, we show that manufacturing of gene-engineered VSTs can be simplified and shortened by combining IFN-γ cytokine capture and retroviral transduction. Our process is scalable, amenable to the use of non-viral gene delivery systems, and yields highly multifunctional T cells with both anti-viral and anti-tumor activity. Clinical translation of our approach can be envisioned in a clinical trial with the goal to prevent or treat viral infection and malignant relapse in patients after allogeneic stem cell transplant.

## Data Availability Statement

The raw data supporting the conclusions of this article will be made available by the authors upon reasonable request.

## Ethics Statement

Ethical review and approval was not required for the study on human participants in accordance with the local legislation and institutional requirements. Written informed consent for participation was not required for this study in accordance with the national legislation and the institutional requirements.

## Author Contributions

GB designed research, performed experiments, analyzed, and interpreted results and wrote the manuscript. CA designed research, supervised the entire study, analyzed and interpreted results and wrote the manuscript. Both authors contributed to the article and approved the submitted version.

## Funding

The work was supported by a Leukemia & Lymphoma Society Translational Research Program Grant (6490-16), the Lausanne University Hospital (CHUV) and the University of Lausanne, Switzerland, all to CA. Open access funding was provided by the University of Lausanne.

## Conflict of Interest

CA and GB receive licensing fees from Immatics. GB is a current employee of Immatics. CA has patents and pending patent applications in the field of engineered T-cell therapies. GB has pending patent applications in the field of engineered T cell therapies.

## Publisher’s Note

All claims expressed in this article are solely those of the authors and do not necessarily represent those of their affiliated organizations, or those of the publisher, the editors and the reviewers. Any product that may be evaluated in this article, or claim that may be made by its manufacturer, is not guaranteed or endorsed by the publisher.
