# Erratum: Rapid generation of TCR and CD8αβ transgenic virus specific T cells for immunotherapy of leukemia

**DOI:** 10.3389/fimmu.2023.1185223

**Published:** 2023-03-24

**Authors:** Frontiers Production Office

**Affiliations:** Frontiers Media SA, Lausanne, Switzerland

**Keywords:** immunotherapy, virus-specific T cells, cytokine capture, transgenic TCR, transgenic CD8, engineered T cells, interferon-gamma

An omission to the funding section of the original article was made in error. The following sentence has been added: “Open access funding was provided by the University of Lausanne”.

The original version of this article has been updated.

